# Protective Role of Oxycodone in Myocardial Oxidative Stress and Mitochondrial Dysfunction Induced by Ischemia‐Reperfusion

**DOI:** 10.1002/jbt.70151

**Published:** 2025-01-26

**Authors:** Yongzheng Jiang, Hua He, Xinwei Jia

**Affiliations:** ^1^ The People's Hospital of Jiawang District of Xuzhou City Xuzhou China; ^2^ Department of Cardiology Affiliated Hospital of Hebei University Baoding China

**Keywords:** membrane potential, mitochondria, oxidative stress, reperfusion injury, signal transduction

## Abstract

Ischemia‐reperfusion (I/R) injury is a significant clinical problem impacting the heart and other organs, such as the kidneys and liver. This study explores the protective effects of oxycodone on myocardial I/R injury and its underlying mechanisms. Using a myocardial I/R model in Sprague‐Dawley (SD) rats and an oxygen‐glucose deprivation/reoxygenation (OGD/R) model in H9c2 cells, we administered oxycodone and inhibited AMP‐activated protein kinase (AMPK) with Compound C (C.C). Our results showed that oxycodone significantly reduced lactate dehydrogenase (LDH) release and reactive oxygen species (ROS) production while stabilizing mitochondrial membrane potential (MMP). Western blot and RT‐qPCR analyzes confirmed that oxycodone enhances AMPK phosphorylation and upregulates the expression of Silent Information Regulator 1 (SIRT1) and Peroxisome Proliferator‐Activated Receptor γ Coactivator 1α (PGC‐1α), thereby protecting myocardial cells. These findings suggest that oxycodone exerts significant protective effects against I/R injury by activating the AMPK pathway, offering new potential therapeutic targets for myocardial protection.

## Graphical abstract

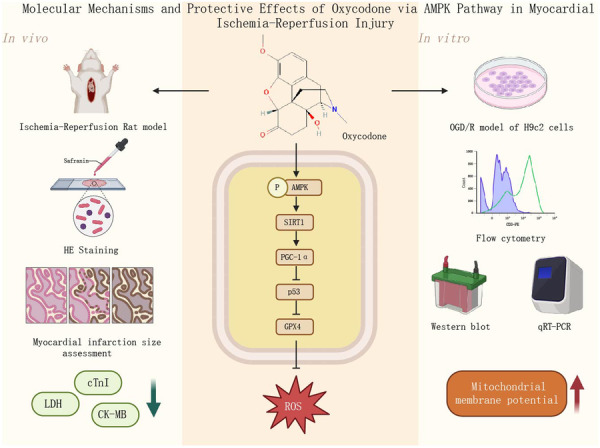

**Abbreviations**
ABI: Applied Biosystems
AMPK: AMP‐activated Protein Kinase
ANOVA: Analysis of Variance
CCK‐8: Cell Counting Kit‐8
cDNA: Complementary DNA
CK‐MB: Creatine Kinase‐MB
cTnI: Cardiac Troponin I
C.C: Compound C
DMEM: Dulbecco's Modified Eagle Medium
EP: Eppendorf
H&E: Hematoxylin and Eosin
IOD: Integrated optical density
I/R: Ischemia‐Reperfusion
LAD: Left Anterior Descending Coronary Artery
LDH: Lactate Dehydrogenase
LSCM: Laser Scanning Confocal Microscopy
MDA: Malondialdehyde
MMP: Mitochondrial Membrane Potential
MP: Myocardial Protection
OD: Optical Density
OGD: Oxygen‐Glucose Deprivation
ROS: Reactive Oxygen Species
SD: Sprague‐Dawley
SD: Sprague‐Dawley
TJ: Tight Junction
TTC: 2,3,5‐Triphenyltetrazolium Chloride
WB: Western Blot

## 1 Introduction

Ischemia‐reperfusion (I/R) injury is a complex pathological process commonly observed in various acute conditions, affecting multiple vital organs, including the heart, kidneys, liver, and brain, with significant implications for patient prognosis [1, 2]. The mechanisms underlying I/R injury are intricate, involving numerous biochemical reactions and cellular signaling pathways [3, 4]. Clinically, I/R injury is closely associated with various acute diseases. For example, in the cardiovascular system, reperfusion following myocardial infarction can lead to further myocardial cell damage, known as myocardial I/R injury, which often results in deteriorated cardiac function and may even progress to heart failure [5, 6]. Similarly, patients with acute kidney failure who experience renal I/R typically undergo rapid renal function decline, leading to severe kidney damage [7, 8]. During the ischemic phase, the interruption of blood supply leads to cellular hypoxia and nutrient deprivation, triggering a cascade of metabolic disturbances and apoptosis. Although restoring the blood supply replenishes oxygen in the reperfusion phase, it also induces severe oxidative stress, inflammatory responses, and calcium overload, exacerbating cellular damage [9, 10]. Consequently, the effective prevention and treatment of I/R injury remain significant challenges in contemporary medicine [11]. Moreover, I/R injury is not confined to specific organs but exhibits systemic effects, further complicating clinical management [12, 13].

In the pathological mechanisms of I/R injury, the AMP‐activated protein kinase (AMPK) signaling pathway is considered a key regulatory factor [14, 15]. As a central regulator of cellular energy balance, AMPK is activated in response to energy metabolism disturbances, promoting energy‐generating pathways while inhibiting energy‐consuming processes [16, 17]. During I/R injury, oxidative stress and energy deficiency‐induced cellular damage make activating the AMPK signaling pathway a potential protective mechanism [18, 19]. Research indicates that AMPK plays a crucial protective role in I/R injury across various organs, including the heart, kidneys, brain, liver, lungs, and intestines, by regulating cellular metabolism, mitigating oxidative stress, maintaining mitochondrial function, and promoting autophagy [20, 21, 22]. Additionally, AMPK enhances cellular stress resistance through interactions with other signaling pathways, such as SIRT1 and PGC‐1α [23, 24]. However, the precise regulatory mechanisms of the AMPK signaling pathway and its comprehensive role in I/R injury require further investigation to identify more specific targets for clinical intervention.

Oxycodone, a commonly used opioid analgesic, is primarily employed for the relief of moderate to severe pain [25]. Its unique analgesic properties and relatively low side effects have led to its widespread clinical use. However, recent studies have increasingly revealed pharmacological actions of oxycodone beyond analgesia, particularly its potential in anti‐inflammatory and cellular protective roles. For instance, oxycodone has demonstrated efficacy in alleviating oxidative stress and inflammatory responses in various models [26, 27], providing a theoretical basis for its application in I/R injury. Preliminary research suggests that oxycodone may exert cellular protective effects by modulating multiple intracellular signaling pathways, including the AMPK pathway [28]. Nonetheless, detailed studies on how oxycodone specifically mediates its effects through the AMPK signaling pathway in myocardial I/R injury remain scant, necessitating further investigation.

This study aims to investigate the effect of oxycodone on oxidative stress and AMPK signaling in myocardial ischemia‐reperfusion injury using a Sprague–Dawley (SD) rat model and H9c2 cells treated with oxygen‐glucose deprivation (OGD) followed by reoxygenation. Western blot and RT‐qPCR will be used to analyze protein expression. Additionally, lactate dehydrogenase (LDH) release, reactive oxygen species (ROS) generation, and mitochondrial membrane potential (MMP) changes will be assessed to explore how oxycodone alleviates I/R injury through the AMPK pathway and its protective role in cardiomyocytes. Through this study, we aim to provide new theoretical insights and potential molecular targets for treating I/R injury, facilitating the development of novel therapeutic strategies and offering more effective clinical treatment options.

## 2 Materials and Methods

### 2.1 Main Reagents and Instruments

Rat cardiomyocytes H9c2 (2‐1) (Cell Bank of the Chinese Academy of Sciences), high‐glucose DMEM (Procell, 4,500 mg/L d‐glucose, 584 mg/L l‐glutamine, 110 mg/L sodium pyruvate), fetal bovine serum (Procell), 350 mL AnaeroPack 15 × 30 cm sealed culture bag (Mitsubishi), oxycodone (Solarbio), compound C (C.C, MCE), 2,3,5‐triphenyltetrazolium chloride (TTC, Sigma), hematoxylin and eosin (H&E) staining solution (Sigma), TRIzol Reagent (Invitrogen), RevertAid RT Kit (Invitrogen), PrimeScript™ RT‐PCR Kit (TaKaRa), Cell Counting Kit‐8 (CCK‐8, ShareBio), lactate dehydrogenase (LDH), creatine kinase‐MB (CK‐MB), and cardiac troponin I (cTnI) assay kits (Nanjing Jiancheng Bioengineering Institute), reactive oxygen species (ROS) detection kit (Labgic), MMP assay kit (JC‐10) (Solarbio), MDA content assay kit (Solarbio), ZO‐1, Claudin‐1, and Occludin antibodies (Abcam), AMPK alpha 1/2, phospho‐AMPK alpha 2, SIRT1, PGC1 alpha, p53, GPX4, GAPDH rabbit polyclonal antibodies, and goat anti‐rabbit IgG HRP (ZEN‐BIO). Applied Biosystems (ABI) PCR System 7500, full‐wavelength microplate reader (BioTek EPOCH), fluorescence inverted microscope (Nikon TI‐S), flow cytometer (BD FACS Calibur), laser scanning confocal microscope (OLYMPUS FV3000). Software: GraphPad Prism (version 9.3.1), FlowJo (version 10.5.3), and Image J (version 1.53c).

### 2.2 Rats

A total of 24 male Sprague‐Dawley (SD) rats weighing 200–300 g (6–8 weeks old) [29, 30] were purchased from Shanghai Jiesijie Laboratory Animal Co. The rats were housed in an air‐conditioned room at 23 ± 2°C with a 12‐h light/dark cycle. They were fed a standard diet with ad libitum access to water. Before the experiment, the rats were acclimatized for 1 week. All animal experiments were approved by the Animal Welfare and Ethical Committee of Hebei University (IACUC‐2019009SR, approved on October 28, 2019).

### 2.3 Induction of Myocardial I/R Injury

Myocardial I/R surgery was performed as described previously [31]. To simulate myocardial I/R injury, we anesthetized the rats intraperitoneally with 40 mg/kg of sodium pentobarbital. After opening the pericardium, the left anterior descending coronary artery (LAD) was exposed and ligated with a 5‐0 silk suture to occlude the vessel. After 30 min of ischemia, the slipknot was loosened to allow 2 h of reperfusion. Ischemia was confirmed by observing changes in the color of the ischemic myocardial tissue, and reperfusion was achieved by releasing the ligature. After 2 h of reperfusion, the animals were euthanized by intraperitoneal injection of 200 mg/kg sodium pentobarbital. Blood samples and heart tissues were collected for subsequent experiments.

### 2.4 Grouping and Drug Administration

The SD rats were randomly divided into control, Sham, I/R, and I/R + Oxycodone (6 rats per group). Rats in the Oxycodone group received an intravenous injection of 0.5 mg/kg of oxycodone 30 min before surgery [32]. The other groups received an intravenous injection of saline at the same time.

### 2.5 Assessment of Myocardial Infarct Size

Myocardial infarct size was assessed using TTC staining [33]. After being rinsed three times with saline, the heart tissue was cut into five slices, each 2 mm thick. The slices were stained with 1% TTC solution (T8877; Sigma‐Aldrich) at 37°C for 15 min. The infarct area was identified and quantified using Image J software (Version 1.52 r; National Institutes of Health, Bethesda, MA, USA).

### 2.6 Determination of Serum Levels of LDH, CK‐MB, and CTnI

Blood samples were centrifuged at 3000 rpm for 20 min at 4°C to assess myocardial cell injury to obtain serum. The levels of LDH (A020‐1‐2), CK‐MB (H197‐1‐1), and cTnI (H149‐2) [34] in the serum were measured using commercial assay kits provided by Nanjing Jiancheng Bioengineering Institute (Nanjing, China).

### 2.7 H&E Staining

The heart tissue was washed thrice with saline, fixed in 4% paraformaldehyde for 24 h, and embedded in paraffin. The sections (5 μm thick) were baked at 65°C for 1 h. Dewaxing and rehydration were performed by sequentially placing the sections in xylene I for 20 min, xylene II for 20 min, absolute ethanol I for 5 min, absolute ethanol II for 5 min, 95% ethanol for 5 min, 85% ethanol for 5 min, 75% ethanol for 5 min, ddH2O I for 5 min, and ddH2O II for 5 min. Hematoxylin staining was performed for 3–5 min, followed by washing with running water for 10 min and differentiation using a solution of 1% hydrochloric acid ethanol (99 mL 75% ethanol and 1 mL concentrated hydrochloric acid). The sections were blued with 1% ammonia water and washed again with running water for 10 min. Eosin staining was performed for 5 min after dehydration in 85% and 95% ethanol for 5 min each. Dehydration and clearing were carried out by placing the sections in absolute ethanol I for 5 min, absolute ethanol II for 5 min, absolute ethanol III for 5 min, xylene I for 10 min, and xylene II for 10 min. Finally, the sections were mounted with neutral balsam. Imaging was performed with an Olympus optical microscope [35].

### 2.8 Immunohistochemical Staining

Following standard histological procedures, fixed biopsy specimens were embedded in paraffin blocks (≤ 72 h). The tissue sections were rehydrated through a graded series of ethanol solutions, and endogenous peroxidase activity was blocked with 5% hydrogen peroxide. Antigen retrieval was performed (6 min × 2 cycles), followed by blocking with 6% serum for 60 min. The sections were then incubated overnight (18 h) at 4°C with p‐AMPKα (Thr172) antibody (1:100, Boster Biological Technology, China). The sections were subsequently incubated with horseradish peroxidase‐conjugated goat anti‐rabbit IgG (1:200) at 37°C for 1 h and stained with diaminobenzidine (DAB) for 5‐30 s. Images were captured using an optical microscope with the digital Leica QWin V3 system (Leica Ltd, Germany). For each sample, three slides were evaluated blindly, with three fields of view per slide. The immunostaining integrated optical density (IOD) was quantitatively analyzed using Image‐Pro Plus version 6.0 (Media Cybernetics, USA).

### 2.9 Determination of Oxygen‐Glucose Deprivation (OGD) and Reoxygenation Times

When H9c2 cells reached the logarithmic growth phase, they were cultured in high‐glucose DMEM containing 1% FBS for 24 h to synchronize the cell cycle to the G0 phase. Cells were then counted and adjusted to a concentration of 5 × 10⁴/mL. Four 96‐well culture plates were selected and labeled for OGD times of 6, 8, 12, and 24 h. The middle seven columns of each plate were selected and labeled for reoxygenation times of 0 h (control group), 2, 4, 6, 12, 18, and 24 h. Each column was allocated 6 samples, with 100 μL of cell suspension added to each well. Six wells in a reserved column were selected, and 100 μL of high‐glucose DMEM containing 10% FBS was added as background detection. The cells were cultured for 12 h in a cell incubator at 37°C with 5% CO₂. Before OGD, 10 μL of CCK‐8 working solution was added to the control group, and the plate was incubated for 2 h in a 37°C incubator. The optical density (OD) values were measured using a microplate reader. After OGD, the original culture medium in each well was replaced with 100 μL of glucose‐free and serum‐free DMEM. Four 15 × 30 cm sealed culture bags were labeled as OGD 6 h, OGD 8 h, OGD 12 h, and OGD 24 h groups. The corresponding culture plates were placed into the labeled sealed bags, with oxygen indicators and 350 mL of AnaeroPack quickly added before sealing.

The sealed bags were placed back into the cell incubator for continued incubation. Successful deoxygenation was indicated when the oxygen indicator in the bag turned red. The corresponding sealed bags were opened after 6, 8, 12, and 24 h of OGD, and the original culture medium was removed. Then, 100 μL of high‐glucose DMEM containing 10% FBS was added to each well for continued culture. At 0, 2, 4, 10, 16, and 22 h of reoxygenation, 10 μL of CCK‐8 working solution was added to each well, followed by a 2‐h incubation in the cell incubator. The OD values of each well were measured using a microplate reader. Background OD values were subtracted for each group, and the data were imported into GraphPad Prism for one‐way analysis of variance (ANOVA) and bar chart analysis.

### 2.10 Determination of Oxycodone Concentration

Based on the data obtained in the previous step, the OGD/R model was established with 12 h of OGD followed by 6 h of reoxygenation. Two 96‐well culture plates were used. Six wells in the middle column were selected for the first plate, with 100 μL of cell suspension added to each well, and labeled as the control group. The second plate was labeled vertically according to the oxycodone concentrations: 0.01, 0.025, 0.05, 0.075, 0.1, and 0.2 mM. Each column was allocated 6 samples, with 100 μL of cell suspension added to each well. Six wells in a reserved column were selected, and 100 μL of high‐glucose DMEM containing 10% FBS was added as background detection. Four hours before OGD, the second plate was pretreated with oxycodone at the indicated concentrations. The culture medium in the second plate was then replaced with 100 μL of glucose‐free and serum‐free DMEM. The plate was placed into a sealed culture bag, and 350 mL of AnaeroPack was quickly added before sealing the bag. The plate was incubated in a cell incubator for 12 h. After incubation, the 96‐well plate was removed from the sealed bag, and the original culture medium in each well was discarded. Then, 100 μL of high‐glucose DMEM containing 10% FBS was added to each well, and the plate was incubated in the cell incubator for an additional 6 h. Next, 10 μL of CCK‐8 working solution was added to each well, followed by a 2‐h incubation in the cell incubator. The background OD values were subtracted for each group, and the data were imported into GraphPad Prism for ANOVA and bar chart analysis.

### 2.11 Experimental Grouping and Establishment of the OGD/R Model

Based on the previous experiment, the optimal concentration of oxycodone was determined to be 0.1 mM, while the recommended concentration of C.C (an AMPK inhibitor) was 10 μM. The experiment was divided into four groups: The control group, the OGD/R group, the OGD/R+Oxy group, and the OGD/R + Oxy + C.C group. First, the cell suspension density was adjusted to 5.0 × 10⁵/mL, and H9c2 cells were seeded into six‐well plates for each group, with 2 mL of cell suspension added to each well. The cells were incubated at 37°C with 5% CO₂ in a cell incubator for 12 h. In the OGD/R + Oxy group and OGD/R+Oxy+C.C group, cells were pretreated with 0.1 mM oxycodone 4 h before the onset of OGD, while the OGD/R + Oxy + C.C group was additionally pretreated with 10 μM C.C for 4 h. The original culture medium was then replaced with 1 mL of glucose‐free and serum‐free DMEM, and the plates were placed in sealed culture bags and incubated with AnaeroPack. After 12 h of OGD, the plates were removed, and the medium was replaced with 2 mL of high‐glucose DMEM containing 10% FBS for continued incubation for 6 h. This grouping and modeling method was used for subsequent experiments.

### 2.12 LDH Assay

We collected 200 μL of culture medium from each group after 12 h of OGD and 6 h of reoxygenation for LDH detection. After serially diluting the standard, reagents were sequentially added for the reaction, and the OD value at 450 nm was measured using a microplate reader. A standard curve was plotted, and the standard curve equation was calculated. The 96‐well plate was labeled horizontally with blanks, standards, test samples for each group, and controls, with six replicate wells set for each group vertically. Samples and reagents were added, and the OD values at A450 nm were measured. The standard curve equation Y was derived as the test sample's OD value minus the control's. This equation was used to calculate each group's pyruvate concentration x (μmol/mL). The enzyme activity unit was defined as the amount of enzyme that catalyzes the formation of 1 nmol of pyruvate per minute per milliliter of sample. Thus, LDH activity (U/mL) was calculated as x·10³ ÷ 15 min = 66.7·x. The data were exported to GraphPad Prism for ANOVA and bar chart plotting.

### 2.13 Fluorescence Microscopy

A six‐well culture plate was prepared, and cells were stained with H2DCFDA fluorescent probe diluted at 1:1000. The fluorescence microscope was set up with a 10× eyepiece and a 4× objective lens, and the focus was adjusted under bright‐field illumination. DCF was excited using a high‐pressure mercury lamp and a blue filter, causing it to emit green fluorescence. Fluorescent images were observed and captured using the fluorescence microscope camera. The average fluorescence intensity of the images was analyzed using ImageJ.

### 2.14 Flow Cytometry

A six‐well culture plate was prepared, and cells were stained with H2DCFDA fluorescent probe diluted at 1:1000. The cells from each group were digested, collected, and centrifuged at 3000 rpm for 5 min. The supernatant was carefully discarded, and the cells were resuspended in 300 μL of 1× PBS before being transferred to flow cytometry tubes. The FL1 channel of the flow cytometer was set to excite at 488 nm and measure emission at 530 nm. A total of 10,000 cells were collected for each group. The data were exported to FlowJo for gating and chart analysis [36].

### 2.15 Laser Scanning Confocal Microscopy (LSCM)

Cells from each group were seeded onto 3.5 cm glass‐bottom confocal dishes. The JC‐10 probe was diluted and applied to the cells, and fluorescence was observed under green laser (Oregon Green 488) and red laser (DS‐RED2) excitation. The integrated density and area of the fluorescence were analyzed using ImageJ.

### 2.16 Lipid Peroxidation

Cells from the six‐well culture plates were lysed by sonication and then transferred to 1.5 mL Eppendorf (EP) tubes. The MDA and TBA working solution was mixed with the lysate and boiled for 60 min. After cooling to room temperature, 200 μL of the supernatant from each group was transferred to a 96‐well plate. The OD values were measured at 450, 532, and 600 nm. The MDA content was calculated using the appropriate formula, and the data were exported to GraphPad Prism for ANOVA and bar chart plotting [37].

### 2.17 Western Blot (WB)

Cells were lysed to extract total protein using a mixture of RIPA buffer, PMSF, and phosphatase inhibitors (100:1:1). The protein concentration was quantified using the BCA assay. Primary antibodies were diluted in 5% BSA as follows: ZO‐1 (1:1000), Claudin‐1 (1:1000), Occludin (1:1000), AMPK (1:1000), p‐AMPK (1:1000), SIRT1 (1:1000), PGC‐1α (1:1000), p53 (1:1000), GPX4 (1:1000), and GAPDH (1:5000). Secondary antibodies were diluted 1:20000 in 5% non‐fat milk. A 10% SDS‐PAGE gel was prepared, and 30 μg of protein was loaded into each well. The gel was run at a constant voltage of 80 V for 15 min, followed by 120 V for 80 min. Proteins were transferred to a PVDF membrane at a constant current of 280 mA for 180 min. After transfer, the membrane was blocked with 5% non‐fat milk for 30 min and washed 6 times for 5 min each. The membrane was incubated with the primary antibodies overnight. After incubation, the membrane was washed 6 times for 5 min each and then incubated with the secondary antibodies for 90 min. Following another round of washing (6 times for 5 min each), the membrane was subjected to ECL detection, imaging, fixing, and scanning. The OD of each band was quantified using ImageJ, and the ratios of p‐AMPK to total AMPK and other primary antibodies to GAPDH were calculated. The data were exported to GraphPad Prism for ANOVA and bar chart plotting.

### 2.18 RT‐qPCR

Total RNA was isolated from heart tissue and cells using TRIzol reagent (Invitrogen, Carlsbad, CA, USA), followed by reverse transcription into complementary DNA (cDNA) using the RevertAid RT kit (cat. no. K1691; Invitrogen, Carlsbad, CA, USA). PCR was performed on the ABI PCR System 7500 (Foster City, CA, USA) using the PrimeScript™ RT‐PCR kit (cat. no. RR014B; TaKaRa). The primers used in this study were as follows: ZO‐1, forward 5′‐ACAGTACAGCCAGCCAGTTC‐3′, reverse 5′‐GGCTCAGCAGAGTTTCACCT‐3′; Claudin‐1, forward 5′‐TGGGGCTGATCGCAATCTTT‐3′, reverse 5′‐ACTTAAGGAGCACCCTTCGC‐3′; Occludin, forward 5′‐ATTGAGCCCGAGTGGAAAGG‐3′, reverse 5′‐GAGGTAGCACCACGTTGGAA‐3′; and β‐actin, forward 5′‐CCTTCTTGGGTATGGAATCCTG‐3′, reverse 5′‐CAATGCCTGGGTACATGGTG‐3′. Target gene expression was normalized to β‐actin and calculated using the 2^−ΔΔCt^ method. Hieff qPCR SYBR Green Master Mix was used for quantitative PCR. Each sample was run in duplicate, and the experiment was independently repeated three times.

### 2.19 Statistical Analysis

All experimental data are presented as mean ± standard deviation (SD). Statistical comparisons between two groups or among multiple groups were performed using independent sample t‐tests and ANOVA, depending on the distribution characteristics and the comparison requirements. For results showing significant differences in the ANOVA, Tukey's multiple comparison test was conducted to explore specific group differences further. A *p*‐value of less than 0.05 was considered statistically significant in all statistical tests. All statistical analyzes were conducted using GraphPad Prism 5.0 (GraphPad Software Inc., USA). A *p*‐value of < 0.05 was considered indicative of statistical significance.

## 3 Results

### 3.1 Effects of Oxycodone on the Ischemic Area and Myocardial Function in Rats With Myocardial I/R Injury

**Figure 1:**
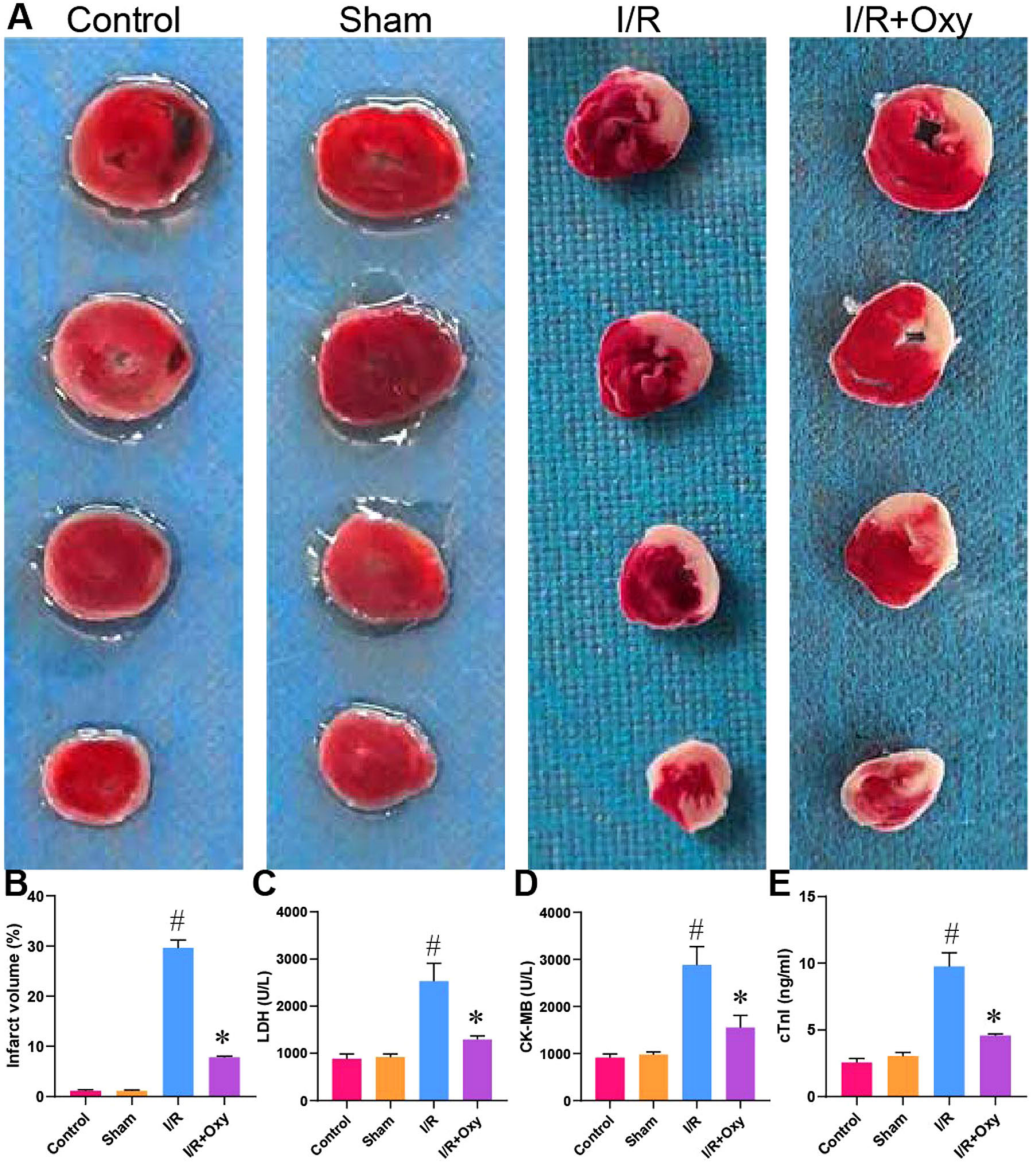
Oxycodone reduces myocardial infarct size and improves cardiac function in rats with myocardial I/R injury. Note: (A) Myocardial infarct size determined by TTC staining; (B) Percentage of myocardial infarct volume; (C) Serum levels of LDH; (D) CK‐MB; (E) cTnI measured using commercial kits. *n* = 3. ^#^ *p* < 0.05 versus control group. *p < 0.05 versus I/R group.

The experiment initially utilized TTC staining to assess the impact of oxycodone on the myocardial ischemic area. There was no significant difference in myocardial infarction area between the sham operation group and the control group. However, compared to the sham operation group, the ischemic area increased following I/R induction, while subsequent administration of oxycodone markedly reduced the ischemic area (Figure 1A,B). cTnI is the gold standard for detecting myocardial I/R injury, LDH is a standard marker of oxidative stress, and CK‐MB is commonly used to diagnose and assess the severity of myocardial infarction and I/R injury [38]. Serum levels of LDH, CK‐MB, and cTnI were measured in rats from each group using commercial assay kits. The results showed that, compared to the sham operation group, I/R induction significantly increased LDH, CK‐MB, and cTnI levels (Figure 1C–E). However, oxycodone treatment suppressed the I/R‐induced elevation of LDH, CK‐MB, and cTnI levels, indicating that oxycodone has a protective effect against myocardial I/R injury. These findings suggest that oxycodone reduces the ischemic area and improves myocardial function in rats with myocardial I/R injury.

### 3.2 The Effects of Oxycodone on Myocardial Tissue Pathological Damage and Endothelial Integrity in Rats With Myocardial I/R Injury

**Figure 2:**
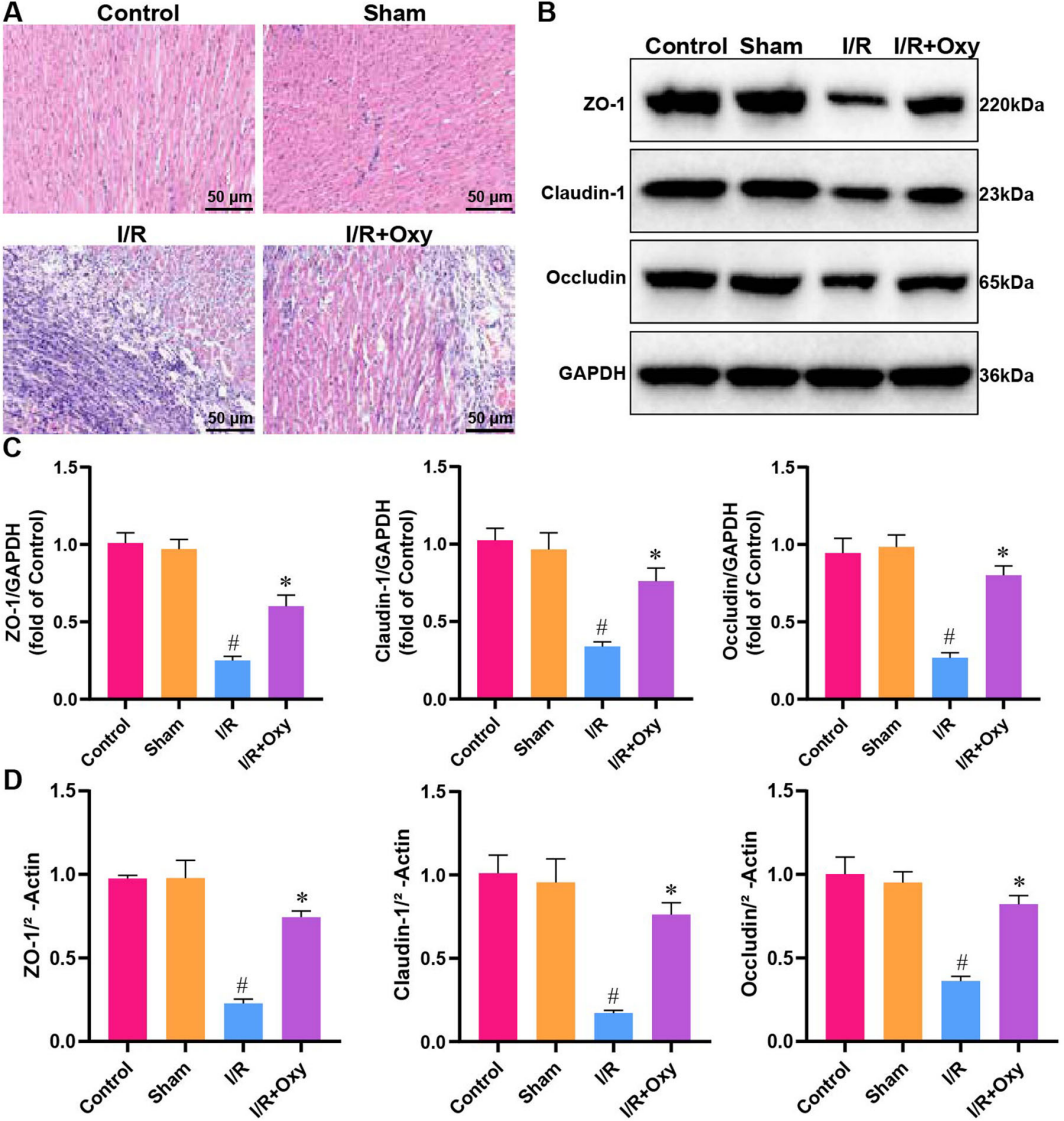
Oxycodone alleviates histopathological damage and enhances endothelial integrity in myocardial I/R‐injured rats. Note: (A) Histopathological changes in myocardial tissue assessed by H&E staining; (B) and (C) Protein expression of ZO‐1, Claudin‐1, and Occludin analyzed by WB; (D) mRNA expression of ZO‐1, Claudin‐1, and Occludin analyzed by RT‐qPCR. *n* = 3. ^#^ *p* < 0.05 versus control group. *p < 0.05 versus I/R group.

The experiment involved H&E staining to assess the impact of oxycodone on the histopathological changes in myocardial tissue across different groups. As shown in Figure 2A, the myocardium in the sham‐operated group exhibited orderly alignment with no significant changes compared to the control group. In contrast, the I/R group displayed disorganized myocardial alignment, evidence of rupture, and inflammatory cell infiltration in the interstitial tissue. However, the oxycodone‐treated group showed a marked reduction in myocardial histopathological damage. Degradation of tight junction (TJ) proteins is a critical factor in endothelial cell injury [39]. The results in Figure 2B–D demonstrate that, compared to the sham‐operated group, I/R stimulation downregulated TJ proteins' protein and mRNA expression, including ZO‐1, Claudin‐1, and Occludin, in myocardial tissue. Oxycodone treatment partially reversed this downregulation. These findings suggest that oxycodone can mitigate myocardial histopathological damage induced by I/R injury and enhance rat endothelial integrity.

### 3.3 The Effects of Oxycodone on the AMPK Pathway in Myocardial Tissue Cells

**Figure 3:**
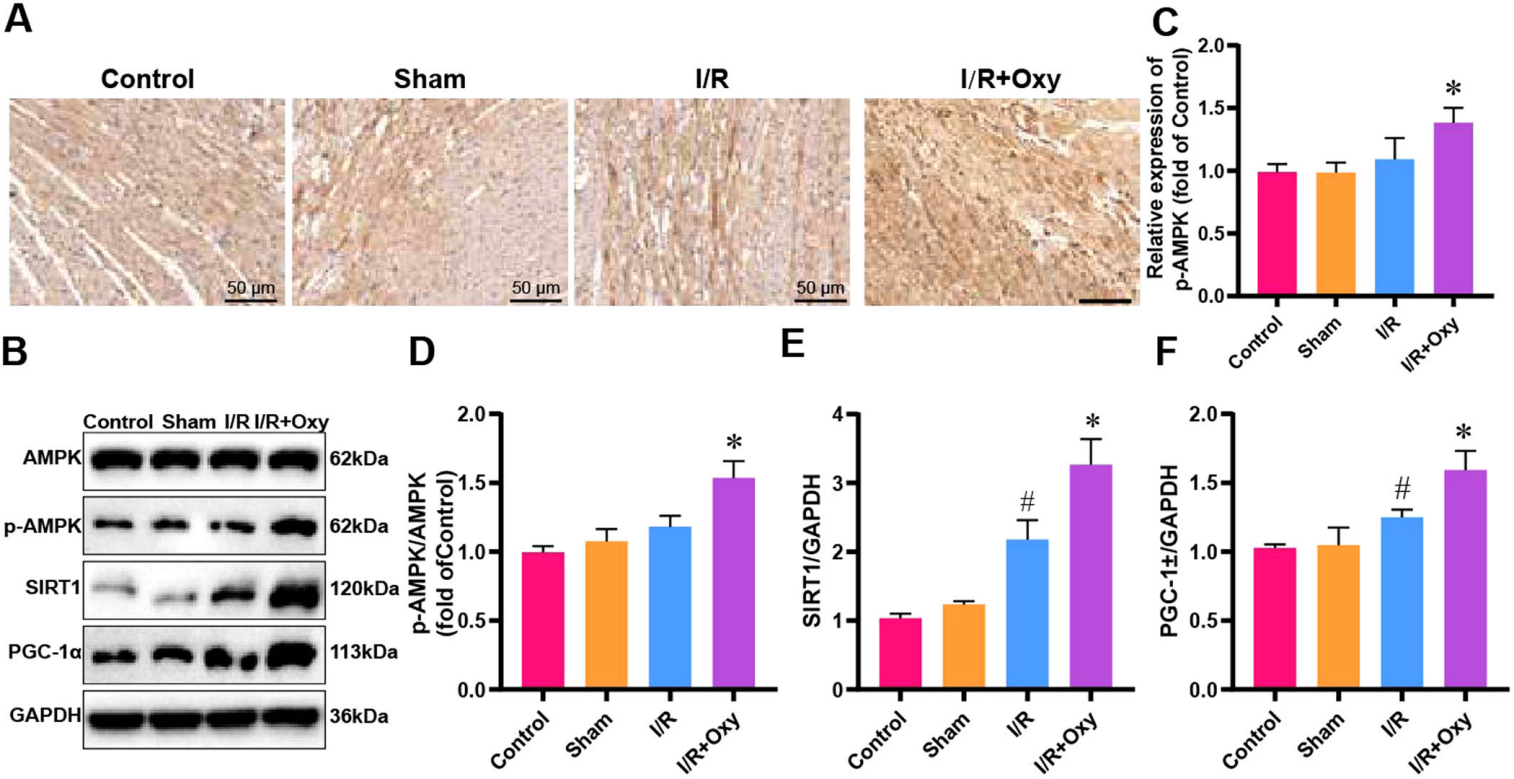
Effects of oxycodone on the AMPK pathway in myocardial tissue. Note: (A, C) Representative immunohistochemical staining images of p‐AMPK‐positive cells in myocardial tissue from different groups, with quantification; (B, D–F) Protein expression levels of AMPK/p‐AMPK, SIRT1, and PGC‐1α in myocardial tissue from different groups analyzed by WB, with quantification. *n* = 3. ^#^ *p* < 0.05 versus control group. *p < 0.05 versus I/R group.

The AMPK pathway is a critical regulator of apoptosis. Previous studies have demonstrated that activating the AMPK pathway can preserve myocardial function and enhance cardiomyocyte viability [40]. In this experiment, immunohistochemical staining was first used to detect the expression of p‐AMPK in myocardial tissues across different groups. The results showed a trend toward increased p‐AMPK‐positive cells in the I/R group compared to the control and sham‐operated groups, though this increase was not statistically significant. However, oxycodone treatment increased the number of p‐AMPK‐positive cells compared to the I/R group (Figure 3A,C). Subsequently, WB analysis was performed to examine the expression levels of key proteins in the AMPK signaling pathway, including AMPK, p‐AMPK, SIRT1, and PGC‐1α, in myocardial tissues from each group. The WB results indicated a trend of increased AMPK phosphorylation and PGC‐1α expression in the OGD/R group compared to the control group, although these changes were not statistically significant. However, there was a significant increase in SIRT1 expression. Compared to the OGD/R group, the OGD/R+Oxy group showed a significant enhancement in the phosphorylation of AMPK, as well as in the expression levels of SIRT1 and PGC‐1α (Figure 3B,D–F). These results suggest that oxycodone effectively activates the AMPK pathway in myocardial cells of rats subjected to I/R injury, thereby protecting against I/R‐induced myocardial damage.

### 3.4 The Effects of Different Durations of OGD and Reoxygenation, as Well as Varying Concentrations of Oxycodone, on the Viability of H9c2 Cells

**Figure 4:**
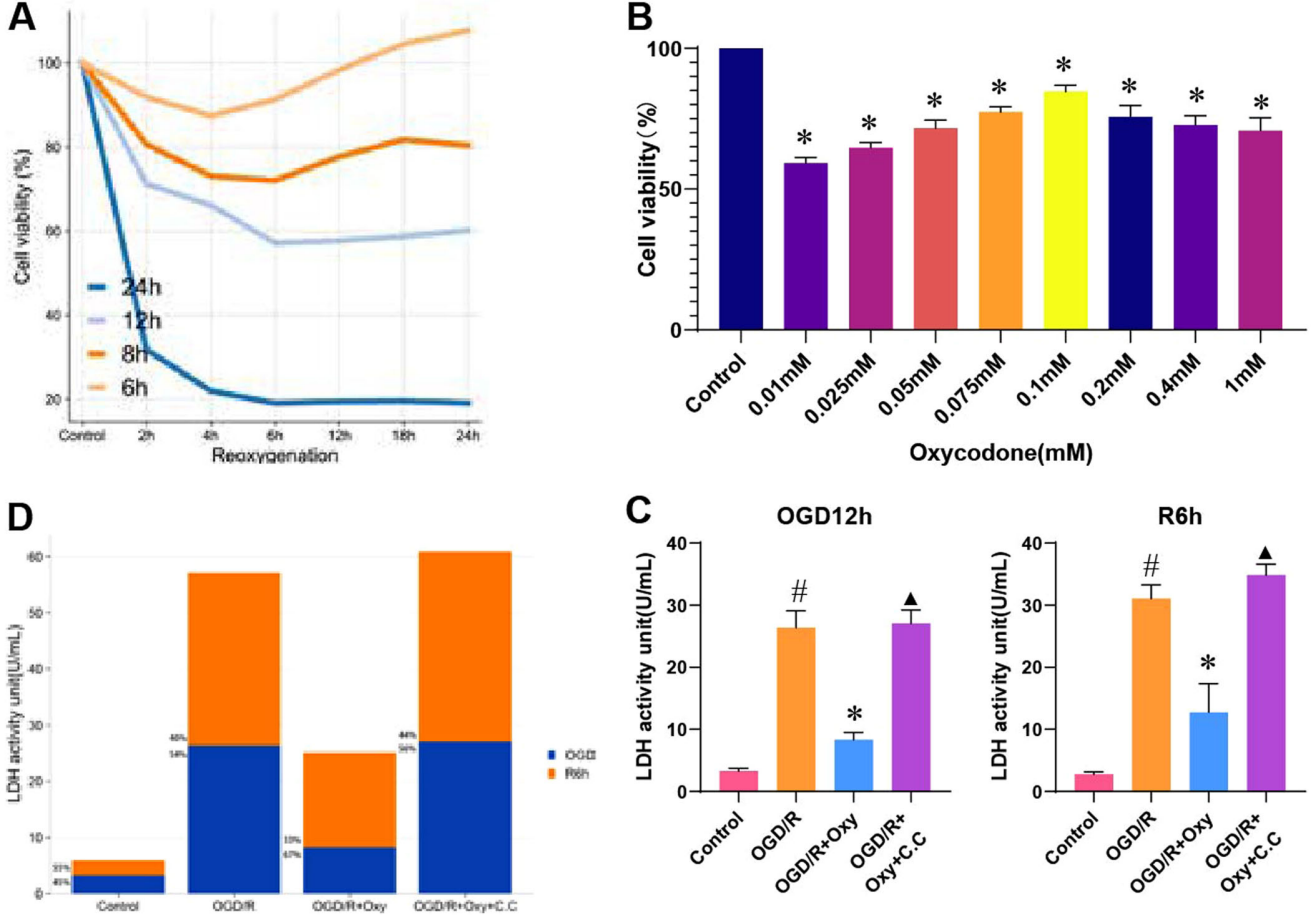
Effects of varying OGD/R durations and oxycodone concentrations on H9c2 cell viability. Note: (A) Impact of different OGD/R durations on H9c2 cell viability; (B) Effect of various oxycodone concentrations on cell viability during OGD/R; (C) LDH release after OGD for 12 h and reoxygenation for 6 h; (D) Total LDH release at different stages of OGD/R. *n* = 3. ^#^ *p* < 0.05 versus control group. *p < 0.05 versus OGD/R group. ▲*p* < 0.05 versus OGD/R + Oxy group.

To further explore whether oxycodone affects I/R‐induced myocardial injury through the regulation of the AMPK signaling pathway, we inhibited AMPK in H9c2 cells using C.C The initial experiment investigated the effects of different durations of OGD and subsequent reoxygenation on H9c2 cell viability. Figure 4A illustrates the changes in H9c2 cell viability following OGD for 6, 8, 12, and 24 h, followed by reoxygenation for 2, 4, 6, 12, 18, and 24 h. The results indicate that H9c2 cell viability decreased slightly after short‐term OGD and reoxygenation, with gradual recovery observed as reoxygenation time increased. However, after prolonged OGD, cell viability declined, and the extent of recovery during reoxygenation was notably limited.

The experiment further investigated the protective effects of various concentrations of oxycodone on H9c2 cells subjected to OGD for 12 h, followed by 6 h of reoxygenation (OGD 12 h + R 6 h). The results demonstrated that as the concentration of oxycodone increased from 0.01 mM, cell viability gradually improved. At a concentration of 0.1 mM, cell viability reached 84.64% ± 2.20%, exhibiting the most significant protective effect (*p* < 0.01). However, with further increases in oxycodone concentration, cell viability began to decline (*p* < 0.01) (Figure 4B). These findings suggest that 0.1 mM is the optimal concentration of oxycodone. Considering both the time factors and cell viability levels, using a 0.1 mM concentration of oxycodone in subsequent experiments is ideal when cell viability stabilizes around 60% after 12 h of OGD and 6 h of reoxygenation. It provides a suitable experimental framework for exploring the protective effects of oxycodone.

### 3.5 Inhibition of AMPK Reverses the Protective Effects of Oxycodone on H9c2 Cells Under Ogd/R‐Induced Injury

LDH release is a critical indicator for assessing cell damage and necrosis in the OGD/R experimental model, which aids in understanding and evaluating the effectiveness of therapeutic strategies to alleviate or prevent I/R injury [41]. Figure 4C illustrates the amount of LDH released by H9c2 cells during 12 h of OGD and 6 h of reperfusion. Figure 4D shows the total LDH released during the entire OGD/R process and the amounts released during OGD and reperfusion separately. In the OGD/R+Oxy group, the LDH release was 8.32 ± 1.14 U/L (*p* < 0.01) during the OGD phase, 12.75 ± 4.6 U/L (*p* < 0.01) during the reperfusion phase, and the total release was 21.08 ± 5.72 U/L (*p* < 0.01). These values were lower than those in the OGD/R group (OGD: 26.37 ± 2.75 U/L, reperfusion: 12.75 ± 4.6 U/L, total: 57.42 ± 4.72 U/L, *p* < 0.01) and the OGD/R+Oxy+C.C group (OGD: 27.06 ± 2.16 U/L, reperfusion: 34.85 ± 1.76 U/L, total: 61.92 ± 3.84 U/L, *p* < 0.01). In all groups except the control, LDH release during the OGD phase was higher than during the reperfusion phase. These results suggest that oxycodone treatment effectively protects H9c2 cells from OGD/R‐induced injury, and the intervention of C.C can reverse this protective effect.

### 3.6 Effects of Oxycodone on ROS and Lipid Peroxidation Levels in H9c2 Cells Induced by Ogd/R

**Figure 5:**
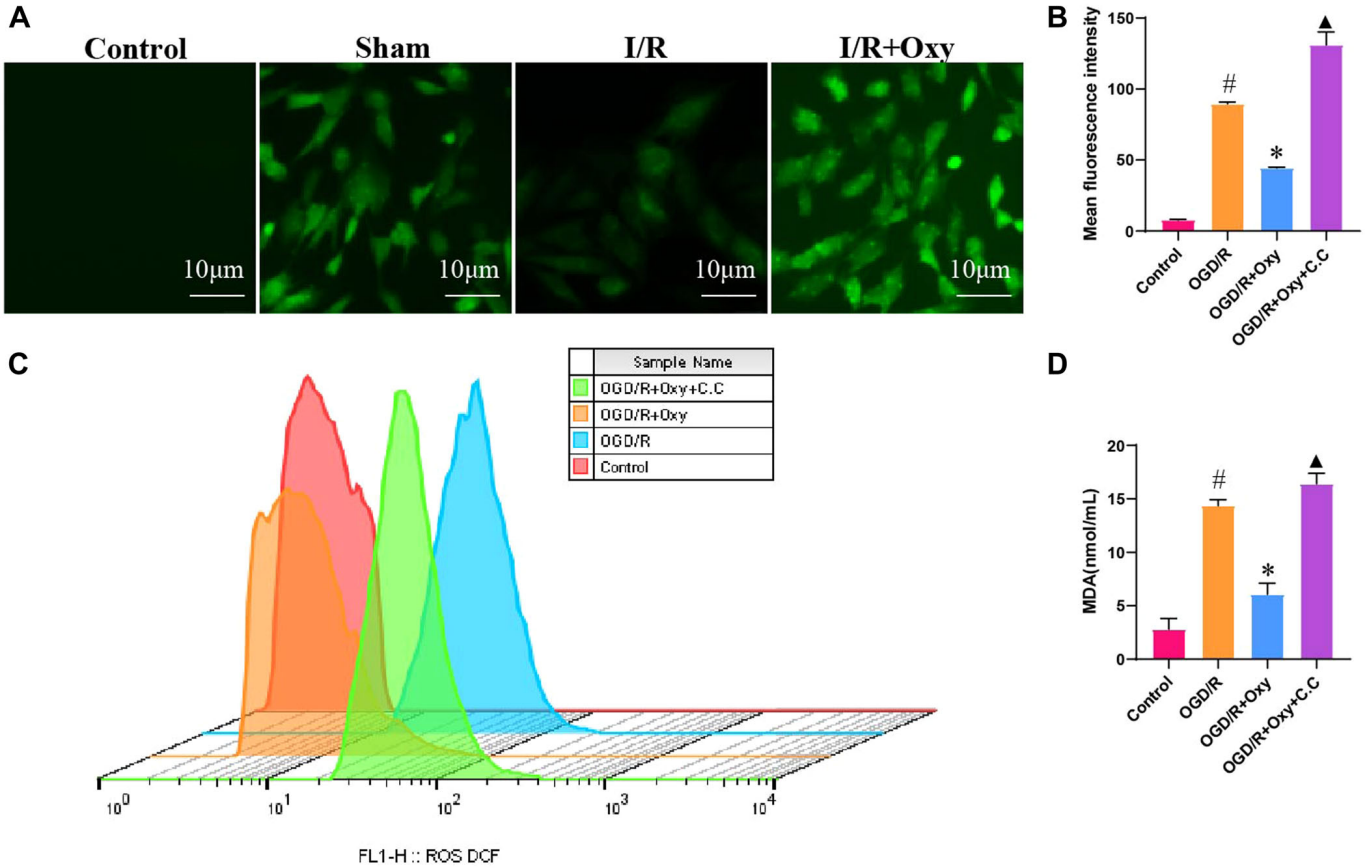
Effects of oxycodone on ROS levels and lipid peroxidation in OGD/R‐induced H9c2 cells. Note: (A, B) Representative images of ROS generation in different groups labeled with the fluorescent probe DCF, with quantification of average fluorescence intensity, *n* = 3; (C) Distribution of ROS‐labeled cells in different groups analyzed by flow cytometry; (D) MDA levels in different groups, *n* = 3. ^#^ *p* < 0.05 versus control group. *p < 0.05 versus OGD/R group. ▲▲*p* < 0.01 versus OGD/R + Oxy group.

Oxidative stress impairs the structure and function of cardiomyocytes through the excessive production of ROS, leading to pathological conditions such as myocardial ischemia and reperfusion injury [41]. This study labeled and quantitatively analyzed ROS generation in H9c2 cells across different experimental groups using the DCF fluorescence probe (Figure 5A,B). Fluorescence microscopy images revealed weak green fluorescence and indistinct cell contours in the control group. In contrast, the OGD/R group exhibited enhanced green fluorescence in the cytoplasm, with clearly defined and varied cell contours. Notably, in the OGD/R + Oxy group, the number of stained cells and the fluorescence intensity were reduced, with the cells maintaining an intact, spindle‐shaped morphology. However, in the OGD/R+Oxy+C.C group, the protective effect of oxycodone was inhibited, as indicated by increased fluorescence intensity and a higher number of stained cells compared to the OGD/R group, along with abnormal cell morphology. Quantitative fluorescence analysis demonstrated that the mean fluorescence intensity in the OGD/R + Oxy group was lower than in the OGD/R and OGD/R + Oxy + C.C groups, with statistical significance (*p* < 0.01). Further analysis of ROS generation via flow cytometry (Figure 5C) showed the following order of peak fluorescence intensity: OGD/R + Oxy + C.C group > OGD/R group > OGD/R + Oxy group > control group. Regarding the peak cell count, the order was: OGD/R + Oxy + C.C group > OGD/R group > control group > OGD/R + Oxy group. These findings indicate that oxycodone reduced ROS production, and this effect could be reversed by inhibition. Finally, malondialdehyde (MDA) levels were measured in the different groups (Figure 5D). The data showed that MDA levels in the OGD/R group were higher than in the control group, while oxycodone treatment reduced OGD/R‐induced MDA levels (*p* < 0.01). However, MDA levels in the OGD/R + Oxy + C.C group were higher than in the OGD/R + Oxy group.

These results suggest that OGD/R treatment increases ROS and MDA production in H9c2 cells, while oxycodone effectively reduces these oxidative stress markers. Furthermore, the inhibition of AMPK can reverse the antioxidative effects of oxycodone.

### 3.7 The Impact of Oxycodone on MMP in OGD/R‐Induced H9c2 Cells

**Figure 6:**
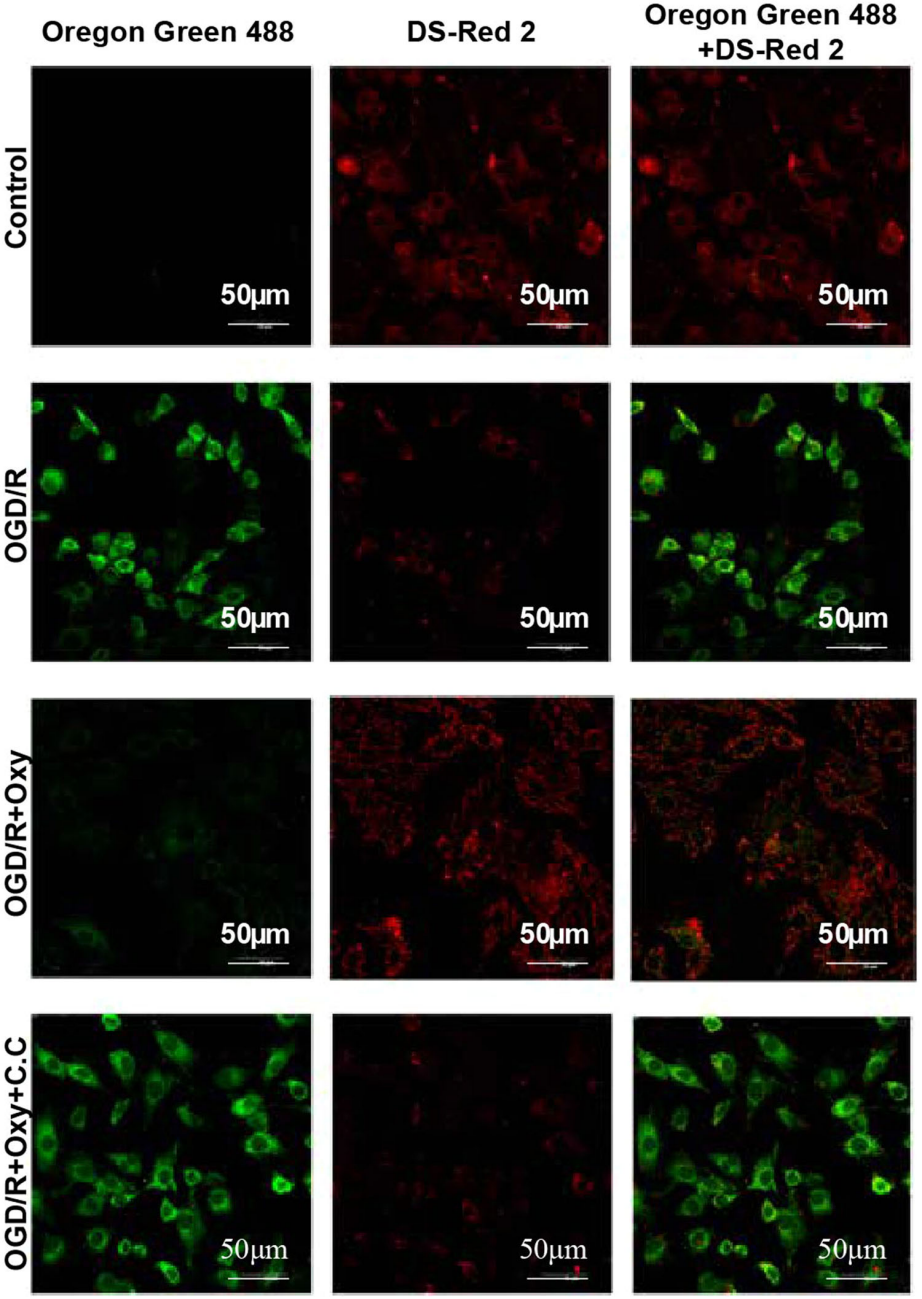
Effects of oxycodone on MMP changes in OGD/R‐induced H9c2 cells. Note: Representative images of mitochondrial fluorescence in different groups under Oregon Green 488 and DS‐RED2 laser excitation.

Cardiac function is highly dependent on mitochondrial oxidative metabolism and quality control, with mitochondrial dysfunction leading to oxidative stress [42]. In this study, changes in MMP in different groups of cells were observed using LSCM with JC‐10 staining. Figure 6 illustrates the mitochondrial fluorescence in each group under green laser (Oregon Green 488) and red laser (DS‐Red2) excitation, observed via LSCM. Table 1 presents the fluorescence distribution area and integrated density analyzed using ImageJ. The results indicate that the control and OGD/R+Oxy groups primarily exhibited red fluorescence. The total red fluorescence intensity was (1045.54 ± 60.48) × 10⁴ in the control group and (426.86 ± 6.93) × 10⁴ in the OGD/R + Oxy group. In contrast, the OGD/R group showed a total red fluorescence intensity of (31.38 ± 1.06) × 10⁴, while the OGD/R + Oxy + C.C group exhibited (262.77 ± 58.46) × 10⁴. These findings demonstrate that the MMP in the OGD/R and OGD/R + Oxy + C.C groups was lower than in the control and OGD/R + Oxy groups. Oxycodone ameliorated the OGD/R‐induced decline in MMP. However, when oxycodone was co‐administered with C.C in the OGD/R + Oxy + C.C group, the improvement in MMP, although present, was still lower than that in the untreated OGD/R control group and the oxycodone‐only group. It suggests that C.C inhibits the protective effects of oxycodone. These results underscore the potential utility of oxycodone in mitigating OGD/R‐induced mitochondrial dysfunction and highlight the inhibitory role of the AMPK inhibitor C.C in this process.

**Table 1:** The area and integrated density of fluorescence distribution (±).

		Integrated Density	Area
Green	Control	(15.13 ± 0.28) × 10^4■^	(1.61 ± 0.55) × 10^3▲^
	OGD/R	(1410.04 ± 162.88) × 10^4^ * ^▲^	(196.91 ± 6.04) × 10^3^ * ^▲^
	OGD/R+Oxy	(175.63 ± 6.22) × 10^4^ #	(33.13 ± 0.95) ×10^3^ *
	OGD/R+Oxy+C.C	(2203.82 ± 6.68) × 10^4^ * ^▲^	(302.72 ± 11.06) × 10^3^ * ^▲^
Red	Control	(1045.54 ± 60.48) × 10^4▲^	(142.52 ± 3.76) × 10^3▲^
	OGD/R	(31.38 ± 1.06) × 10^4^ * ^▲^	(1.25 ± 0.69) × 10^3^ * ^▲^
	OGD/R+Oxy	(426.86 ± 6.93) × 10^4^ *	(16.03 ± 0.77) × 10^3^ *
	OGD/R+Oxy+C.C	(262.77 ± 58.46) × 10^4^ * ^▲^	(20.53 ± 0.70) × 10^3^ * ^■^

*Note:* Compared with the Control group; Compared with the OGD/R + Oxy group,■: *p* > 0.05; ▲: *p* < 0.01

^#^ *p* > 0.05.

* *p* < 0.01.

### 3.8 The Effects of Oxycodone on the AMPK Pathway in H9c2 Cells

**Figure 7:**
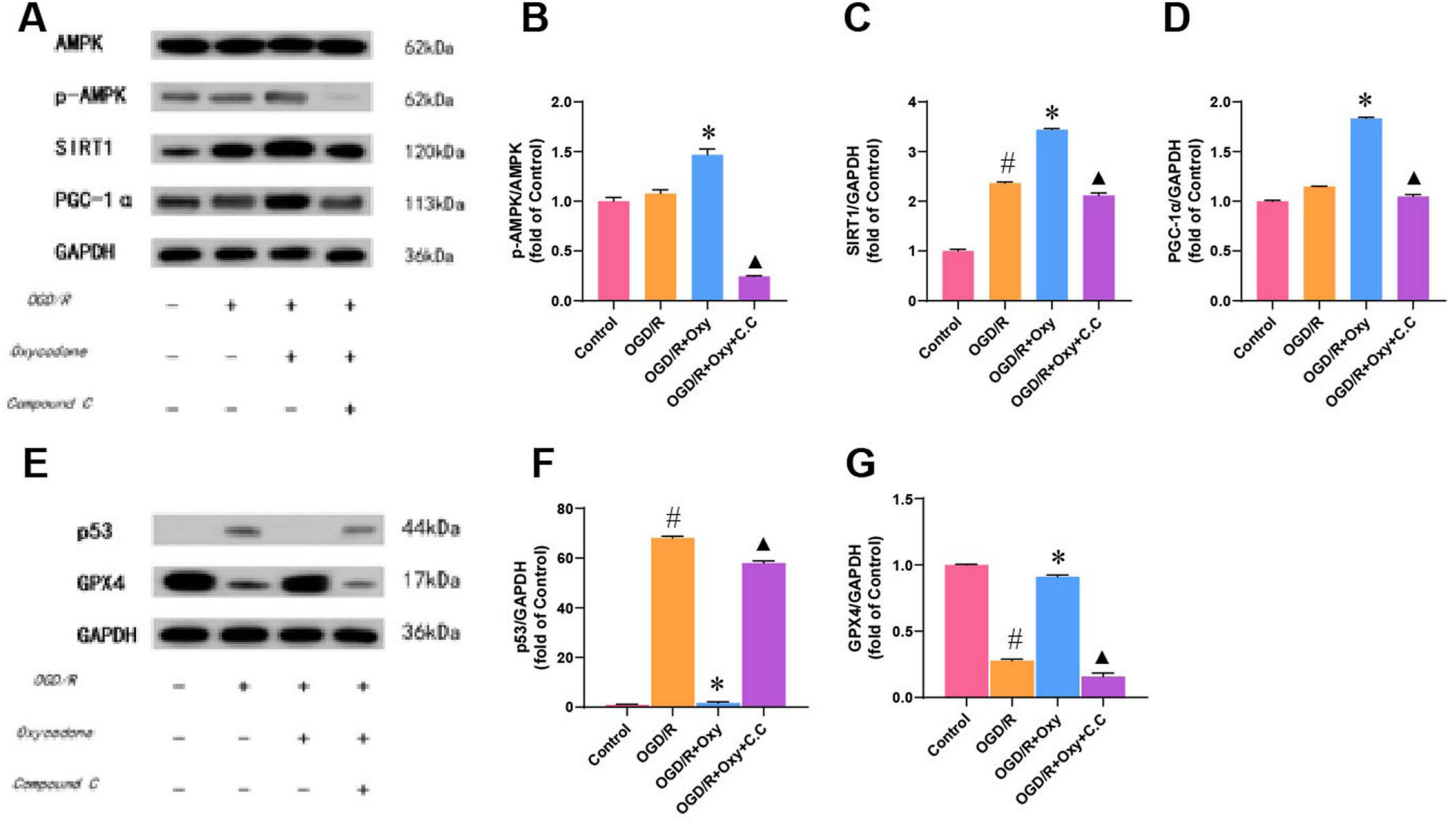
Effects of oxycodone on the AMPK signaling pathway in H9c2 cells. Note: (A–D) Protein expression levels of AMPK, p‐AMPK, SIRT1, and PGC‐1α in different groups analyzed by WB, with quantification; (E–G) Additional quantification of AMPK, p‐AMPK, SIRT1, and PGC‐1α protein levels in various groups. *n* = 3. ^#^ *p* < 0.05 versus control group. *p < 0.05 versus OGD/R group. ▲*p* < 0.05 versus OGD/R + Oxy group.

The experiment investigated the expression levels of AMPK, p‐AMPK, SIRT1, and PGC‐1α proteins in different groups of H9c2 cells using WB analysis. The results indicated that compared to the control group, the OGD/R group exhibited an upward trend in AMPK phosphorylation and PGC‐1α expression, although these changes were not statistically significant (Figure 7A,B,D). However, there was a significant increase in SIRT1 expression in the OGD/R group (Figure 7A,C). In contrast, the OGD/R+Oxy showed a significant increase in the phosphorylation of AMPK, as well as in the expression of SIRT1 and PGC‐1α proteins compared to the OGD/R group. C.C effectively reversed these changes in the OGD/R+Oxy+C.C group (Figure 7A–D). These findings suggest that oxycodone activates the AMPK pathway in H9c2 cells, and this activation is effectively reversed by the AMPK inhibitor C.C. The AMPK pathway is known to influence oxidative stress via p53 and GPX4 [43, 44]. The expression levels of p53 and GPX4 proteins were also examined through WB analysis to investigate this further. Compared to the control group, the OGD/R group showed a significant increase in p53 protein levels and a significant decrease in GPX4 protein levels (Figure 7E–G). However, in the OGD/R + Oxy group, p53 levels were reduced, while GPX4 levels were increased compared to the OGD/R group. C.C again reversed these effects in the OGD/R + Oxy + C.C group (Figure 7E–G). Overall, the results indicate that oxycodone activates the AMPK pathway, leading to decreased p53 expression and increased GPX4 expression, thereby reducing oxidative stress. This effect is counteracted by the AMPK inhibitor C.C.

## 4 Discussion

This study is the first to reveal the protective mechanism by which oxycodone mitigates myocardial I/R injury by activating the AMPK signaling pathway. The experimental results demonstrated that oxycodone treatment led to a marked reduction in LDH release and ROS production and the stabilization of MMP, indicating its strong cytoprotective effects. Furthermore, WB analysis showed that oxycodone promoted AMPK phosphorylation and upregulated the expression of SIRT1 and PGC‐1α. These findings suggest that the AMPK signaling pathway plays a crucial role in the cardioprotective effects induced by oxycodone. Compared to previous studies, this research provides a deeper understanding of oxycodone's cytoprotective mechanisms, particularly in regulating mitochondrial function and oxidative stress through the AMPK signaling pathway.

Compared to previous studies on the effects of oxycodone, this research represents a significant breakthrough in understanding its underlying mechanisms. Earlier studies primarily focused on the analgesic and anti‐inflammatory properties of oxycodone, while this study expands its application to cellular protection [45, 46]. Existing literature has indicated that oxycodone also exerts protective effects on the nervous system and liver, although the specific mechanisms remain unclear [47]. By employing a myocardial I/R model, this study systematically elucidates how oxycodone exerts its protective effects through the AMPK signaling pathway. This discovery offers a new perspective on the multifaceted actions of oxycodone and provides a theoretical foundation for its potential application in cardiovascular diseases.

The role of the AMPK signaling pathway in I/R injury has been extensively studied, particularly in regulating cellular metabolism, combating oxidative stress, and maintaining mitochondrial function [48, 49, 50]. Previous research has primarily focused on the role of AMPK in other organs, such as the liver and kidneys [22, 51]. However, this study centers on the function of AMPK in myocardial I/R injury. Through experimentation, this research confirms that the activation of AMPK enhances cellular antioxidant capacity and mitochondrial function by regulating downstream molecules such as SIRT1 and PGC‐1α. While these findings are consistent with previous studies, this research further clarifies the central role of AMPK in cardioprotection and reveals its specific regulatory mechanisms under the influence of oxycodone.

This study also uncovers a close relationship between oxycodone and the AMPK signaling pathway. The experimental results demonstrate that oxycodone activates AMPK phosphorylation and regulates mitochondrial function through SIRT1 and PGC‐1α. This finding deepens the understanding of the AMPK pathway and provides new insights into the pharmacological actions of oxycodone. Compared to other drugs or molecules that modulate the AMPK pathway, oxycodone exhibits a unique mode of action, particularly in protecting cardiomyocytes. Although the precise molecular mechanisms of oxycodone require further investigation, this study provides preliminary evidence of its potential protective effects mediated through the AMPK pathway.

In the multidimensional exploration of cellular protection mechanisms, this study offers new insights into the role of oxycodone. Given the complex interplay between mitochondrial function, oxidative stress, and autophagy, oxycodone may exert its protective effects through the AMPK pathway and other signaling pathways. While this research primarily focuses on the AMPK signaling pathway, future studies could investigate whether oxycodone modulates cell survival and functions through alternative pathways, such as the PI3K/Akt or NF‐κB signaling pathways. Such investigations would contribute to a more comprehensive understanding of oxycodone's cytoprotective mechanisms and provide broader evidence for its clinical applications.

The findings of this study hold significant clinical implications. As a commonly used opioid in clinical practice, the discovery that oxycodone protects cardiomyocytes through the AMPK signaling pathway could offer a novel approach to treating myocardial I/R injury. Current treatment strategies primarily focus on improving hemodynamics post‐reperfusion and reducing oxidative stress. However, this study introduces the concept of using pharmacological activation of the AMPK pathway to protect cardiomyocytes, presenting a new strategy for cardiovascular disease treatment. Despite these promising findings, the challenge remains in translating these laboratory discoveries into clinical applications. Specifically, determining the optimal dosage and timing for oxycodone administration to maximize its protective effects is crucial for its successful clinical implementation.

Despite the significant findings of this study, certain limitations must be acknowledged. First, the study's conclusions are primarily based on *in vitro*, and animal model experiments, and the applicability of these results to humans requires further validation. Second, although the study highlights the importance of the AMPK pathway in the effects of oxycodone, the specific molecular mechanisms within this pathway need to be explored in greater detail. Additionally, future research could investigate the role of oxycodone in other types of I/R injury, as well as its potential synergistic effects with other drugs. Overall, while this study provides new theoretical support for the use of oxycodone in myocardial protection (MP), further research is necessary to confirm its clinical value and to explore broader applications.

## 5 Conclusion

This study systematically explored the cytoprotective effects of oxycodone in a myocardial I/R injury model and its potential underlying mechanisms. By applying oxycodone to H9c2 cells and a rat I/R model, we found that the drug reduced LDH release, decreased ROS production, and stabilized MMP. Further experiments demonstrated that oxycodone enhances cardiomyocyte protection by activating the AMPK signaling pathway, leading to increased expression levels of SIRT1 and PGC‐1α (Graphical Abstract). These findings provide new theoretical support and molecular targets for the application of oxycodone in the treatment of I/R injury.

This study observed inconsistencies in the regulation of GPX4 and p53 proteins following AMPK pathway activation, which may stem from the multifaceted functions of AMPK and its complex regulatory mechanisms. The literature supports this view: AMPK activation can upregulate the Nrf2/HO‐1 pathway, increasing GPX4 expression and thereby reducing oxidative stress while simultaneously inhibiting p53 stability through SIRT1‐mediated deacetylation, leading to decreased apoptosis [52, 53]. Additionally, the regulation of these proteins by AMPK may vary depending on the duration and intensity of its activation, which could explain the observed discrepancies in protein level changes.

The study found that oxycodone's cytoprotective effects primarily manifest in its ability to inhibit oxidative stress and maintain mitochondrial function. Specifically, oxycodone reduced the generation of ROS and MDA in H9c2 cells following hypoxia/reoxygenation treatment and effectively stabilized the MMP. These results suggest that oxycodone mitigates oxidative stress‐induced cellular damage and enhances cell survival by stabilizing mitochondrial function.

**Figure 8:**
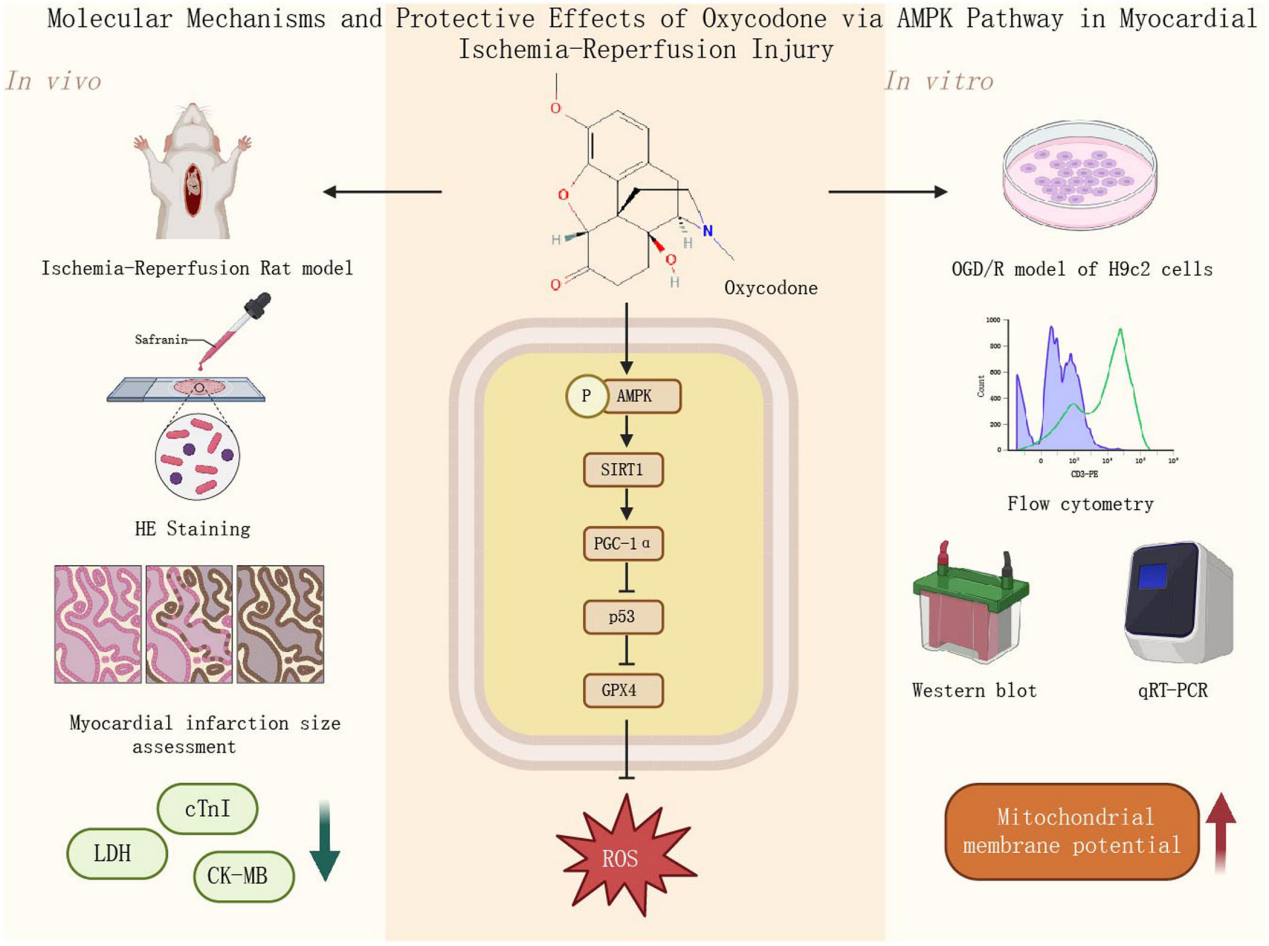
Molecular mechanisms and protective effects of oxycodone via AMPK pathway in myocardial I/R injury.

Furthermore, the inhibition of AMPK was found to reverse the antioxidant effects of oxycodone, further confirming the critical role of the AMPK signaling pathway in oxycodone's protective actions. Overall, this study elucidates the protective mechanism of oxycodone in myocardial I/R injury and provides new insights and potential therapeutic targets for its clinical application. Future research should further explore the protective effects and safety of oxycodone in animal models, uncover additional molecular mechanisms, and, in conjunction with clinical studies, assess the practical efficacy of oxycodone in treating I/R injury, thereby facilitating its translation into clinical practice (Figure 8).

## Author Contributions

Yongzheng Jiang and Xinwei Jia conceived and designed the study. Yongzheng Jiang, Hua He, and Xinwei Jia performed the experiments. Yongzheng Jiang and Hua He analyzed the data. Yongzheng Jiang and Xinwei Jia wrote the manuscript. All authors reviewed and approved the final version of the manuscript.

## Ethics Statement

All animal experiments were approved by the Animal Welfare and Ethical Committee of Hebei University (IACUC‐2019009SR, approved on 28th October, 2019).

## Conflicts of Interest

The authors declare no conflicts of interest.

## Data Availability

The data that support the findings of this study are available from the corresponding author upon reasonable request.

## References

[1] S. Huseyin , A. Reyhancan , U. Halici , et al., “Comparison of the Protective Effects of Vanillic and Rosmarinic Acid on Cardiac Tissue: Lower Limb Ischemia‐Reperfusion Model in Rats.” “Vanilik Ve Rosmarinik Asidin Kalp Dokusu Üzerindeki Koruyucu Etkilerinin Karşılaştırılması: Sıçanlarda Alt Ekstremite Iskemi‐Reperfüzyon Modeli,” Ulusal travma ve acil cerrahi dergisi = Turkish Journal of Trauma & Emergency Surgery: TJTES 30, no. 9 (2024): 619–625, 10.14744/tjtes.2024.12359. 39222491 PMC11622722

[2] M. Zhang , Q. Liu , H. Meng , et al., “Ischemia‐Reperfusion Injury: Molecular Mechanisms and Therapeutic Targets,” Signal Transduction and Targeted Therapy 9, no. 1 (2024): 12, 10.1038/s41392-023-01688-x. 38185705 PMC10772178

[3] D. Patrono and R. Romagnoli , “Postreperfusion Syndrome, Hyperkalemia and Machine Perfusion in Liver Transplantation,” Translational Gastroenterology and Hepatology 4 (September 2019): 68, 10.21037/tgh.2019.08.12. 31620650 PMC6789296

[4] R. O. S. Soares , D. M. Losada , M. C. Jordani , P. Évora , and O. Castro‐e‐Silva , “Ischemia/Reperfusion Injury Revisited: An Overview of the Latest Pharmacological Strategies,” International Journal of Molecular Sciences 20, no. 20 (October 2019): 5034, 10.3390/ijms20205034. 31614478 PMC6834141

[5] M. Chen , X. Li , H. Yang , J. Tang , and S. Zhou , “Hype or Hope: Vagus Nerve Stimulation against Acute Myocardial Ischemia‐Reperfusion Injury,” Trends in Cardiovascular Medicine 30, no. 8 (2020): 481–488, 10.1016/j.tcm.2019.10.011. 31740206

[6] M. Cannistrà , M. Ruggiero , A. Zullo , et al., “Hepatic Ischemia Reperfusion Injury: A Systematic Review of Literature and the Role of Current Drugs and Biomarkers,” International Journal of Surgery 33, no. Suppl 1 (2016): S57–S70, 10.1016/j.ijsu.2016.05.050. 27255130

[7] Q. Y. Zheng , Y. Li , S. J. Liang , et al., “LIGHT Deficiency Attenuates Acute Kidney Disease Development in an in Vivo Experimental Renal Ischemia and Reperfusion Injury Model,” Cell Death Discovery 8, no. 1 (2022): 399, 10.1038/s41420-022-01188-x. 36163116 PMC9512920

[8] C. Li , Y. Yu , S. Zhu , et al., “The Emerging Role of Regulated Cell Death in Ischemia and Reperfusion‐Induced Acute Kidney Injury: Current Evidence and Future Perspectives,” Cell Death Discovery 10, no. 1 (2024): 216, 10.1038/s41420-024-01979-4. 38704372 PMC11069531

[9] J. Li , Y. Zhang , R. Tang , et al., “Glycogen Synthase kinase‐3β: A Multifaceted Player in Ischemia‐Reperfusion Injury and Its Therapeutic Prospects,” Journal of Cellular Physiology 239, no. 9 (2024): e31335, 10.1002/jcp.31335. 38962880

[10] H. J. Chi , M. L. Chen , X. C. Yang , et al., “Progress in Therapies for Myocardial Ischemia Reperfusion Injury,” Current Drug Targets 18, no. 15 (2017): 1712–1721, 10.2174/1389450117666160401120308. 27033199

[11] J. Wang , J. Sun , H. Yu , C. Hu , J. Wu , and C. Hu , “Donor Inhalation of Nebulized Dexmedetomidine Alleviates Ischemia‐Reperfusion Injury in Rat Lung Transplantation,” Pharmacology 109, no. 5 (2024): 293–304, 10.1159/000539528. 38806015

[12] P. Huang , C. Qu , Z. Rao , D. Wu , and J. Zhao , “Bidirectional Regulation Mechanism of TRPM2 Channel: Role in Oxidative Stress, Inflammation and Ischemia‐Reperfusion Injury,” Frontiers in Immunology 15 (2024): 1391355, 10.3389/fimmu.2024.1391355. 39007141 PMC11239348

[13] I. Gheitasi , G. Akbari , and F. Savari , “Physiological and Cellular Mechanisms of Ischemic Preconditioning Micrornas‐Mediated in Underlying of Ischemia/Reperfusion Injury in Different Organs,” Molecular and Cellular Biochemistry. Published ahead of print, July 13, 2024, 10.1007/s11010-024-05052-7. 39001984

[14] J. J. Zhao , B. Zhao , X. Bai , S. Zhang , and R. Xu , “Aucubin Promotes Activation of AMPK and Alleviates Cerebral Ischemia/Reperfusion Injury in Rats,” Cell Stress & Chaperones 28, no. 6 (2023): 801–809, 10.1007/s12192-023-01372-7. 37608231 PMC10746661

[15] C. Fu , S. Yu , Z. Liu , J. Wang , P. Liu , and G. Su , “PFKFB2 Inhibits Ferroptosis in Myocardial Ischemia/Reperfusion Injury Through Adenosine Monophosphate‐Activated Protein Kinase Activation,” Journal of Cardiovascular Pharmacology 82, no. 2 (2023): 128–137, 10.1097/FJC.0000000000001437. 37155368

[16] Kaixin Guo and Yan Lu , “Acupuncture Modulates the AMPK/PGC‐1 Signaling Pathway to Facilitate Mitochondrial Biogenesis and Neural Recovery in Ischemic Stroke Rats,” Frontiers in Molecular Neuroscience 17 (2024): 1388759, 10.3389/fnmol.2024.1388759. 38813438 PMC11133568

[17] N. Wang , B. Wang , and E. P. Maswikiti , “Ampk‐A Key Factor in Crosstalk Between Tumor Cell Energy Metabolism and Immune Microenvironment?,” Cell Death Discovery 10, no. 1 (May 2024): 237, 10.1038/s41420-024-02011-5. 38762523 PMC11102436

[18] Y. W. Xu , C. H. Yao , X. M. Gao , et al., “BAK Ameliorated Cerebral Infarction/Ischemia‐Reperfusion Injury by Activating AMPK/Nrf2 to Inhibit TXNIP/NLRP3/Caspase‐1 Axis,” Neuroscience Letters 844 (2025): 138037, 10.1016/j.neulet.2024.138037. 39515657

[19] V. Zizi , M. Becatti , D. Bani , and S. Nistri , “Serelaxin Protects H9c2 Cardiac Myoblasts Against Hypoxia and Reoxygenation‐Induced Damage Through Activation of AMP Kinase/Sirtuin1: Further Insight Into the Molecular Mechanisms of the Cardioprotection of This Hormone,” Antioxidants (Basel, Switzerland) 13, no. 2 (January 2024): 163, 10.3390/antiox13020163. 38397761 PMC10886064

[20] M. D. A. Paskeh , A. Asadi , S. Mirzaei , et al., “Targeting AMPK Signaling in Ischemic/Reperfusion Injury: From Molecular Mechanism to Pharmacological Interventions,” Cellular Signalling 94 (2022): 110323, 10.1016/j.cellsig.2022.110323. 35358642

[21] G. Xu , Y. Ma , J. Jin , and X. Wang , “Activation of AMPK/p38/Nrf2 is Involved in Resveratrol Alleviating Myocardial Ischemia‐Reperfusion Injury in Diabetic Rats as an Endogenous Antioxidant Stress Feedback,” Annals of Translational Medicine 10, no. 16 (2022): 890, 10.21037/atm-22-3789. 36111006 PMC9469109

[22] R. Ding , W. Wu , Z. Sun , and Z. Li , “AMP‐Activated Protein Kinase: An Attractive Therapeutic Target for Ischemia‐Reperfusion Injury,” European Journal of Pharmacology 888 (2020): 173484, 10.1016/j.ejphar.2020.173484. 32798506

[23] M. Entezari , D. Hashemi , A. Taheriazam , et al., “AMPK Signaling in Diabetes Mellitus, Insulin Resistance and Diabetic Complications: A Pre‐Clinical and Clinical Investigation,” Biomedicine & Pharmacotherapy 146 (2022): 112563, 10.1016/j.biopha.2021.112563. 35062059

[24] N. B. Ruderman , X. Julia Xu , L. Nelson , et al., “AMPK and SIRT1: A Long‐Standing Partnership?,” American Journal of Physiology‐Endocrinology and Metabolism 298, no. 4 (2010): E751–E760, 10.1152/ajpendo.00745.2009. 20103737 PMC2853213

[25] J. S. Gimbel , P. Richards , and R. K. Portenoy , “Controlled‐Release Oxycodone for Pain in Diabetic Neuropathy: A Randomized Controlled Trial,” Neurology 60, no. 6 (2003): 927–934, 10.1212/01.wnl.0000057720.36503.2c. 12654955

[26] Y. Xie , C. L. Ge , Z. Y. Zhang , and G. X. Fei , “Oxycodone Inhibits Myocardial Cell Apoptosis After Myocardial Ischemia‐Reperfusion Injury in Rats via RhoA/ROCK1 Signaling Pathway,” European Review for Medical and Pharmacological Sciences 24, no. 11 (2020): 6371–6379, 10.26355/eurrev_202006_21535. 32572934

[27] M. Ji , J. Cheng , and D. Zhang , “Oxycodone Protects Cardiac Microvascular Endothelial Cells Against Ischemia/Reperfusion Injury by Binding to Sigma‐1 Receptor,” Bioengineered 13, no. 4 (2022): 9628–9644, 10.1080/21655979.2022.2057632. 35412431 PMC9161947

[28] J. C. Newman and E. Verdin , “β‐Hydroxybutyrate: A Signaling Metabolite,” Annual Review of Nutrition 37 (2017): 51–76, 10.1146/annurev-nutr-071816-064916. PMC6640868 28826372

[29] Q. Duan and J. Wu , “Dihydroartemisinin Ameliorates Cerebral I/R Injury in Rats via Regulating Vwf and Autophagy‐Mediated SIRT1/FOXO1 Pathway,” Open medicine (Warsaw, Poland) 18, no. 1 (July 2023): 20230698, 10.1515/med-2023-0698. 37415610 PMC10320570

[30] D. Xie , M. Li , K. Yu , H. Lu , and Y. Chen , “Etomidate Alleviates Cardiac Dysfunction, Fibrosis and Oxidative Stress in Rats With Myocardial Ischemic Reperfusion Injury,” Annals of Translational Medicine 8, no. 18 (2020): 1181, 10.21037/atm-20-6015. 33241030 PMC7576070

[31] M. He , Y. Yu , S. Ning , J. Han , and Z. Guo , “Effects of STAT4 on Myocardial Ischemia‑Reperfusion Injury and the Underlying Mechanisms,” Molecular Medicine Reports 30, no. 5 (2024): 197, 10.3892/mmr.2024.13321. 39239743 PMC11391519

[32] Y. Wang , G. Wu , Z. Liu , et al., “Effect of Oxycodone Combined with Ultrasound‐Guided Thoracic Paravertebral Nerve Block on Postoperative Analgesia in Patients With Lung Cancer Undergoing Thoracoscopic Surgery: Protocol for a Randomised Controlled Study,” BMJ Open 13, no. 10 (October 2023): e074416, 10.1136/bmjopen-2023-074416. PMC10582857 37844986

[33] F. Li , H. Zhu , Z. Chang , and Y. Li , “Gentiopicroside Alleviates Acute Myocardial Infarction Injury in Rats by Disrupting Nrf2/NLRP3 Signaling,” Experimental Biology and Medicine 248, no. 14 (2023): 1254–1266, 10.1177/15353702231199076. 37850391 PMC10621478

[34] J. Lu , J. Wang , K. Han , and N. Li , “Nicorandil Regulates Ferroptosis and Mitigates Septic Cardiomyopathy via TLR4/SLC7A11 Signaling Pathway,” Inflammation 47, no. 3 (2024): 975–988, 10.1007/s10753-023-01954-8. 38159178 PMC11147835

[35] A. H. Fischer , K. A. Jacobson , J. Rose , and R. Zeller , “Hematoxylin and Eosin Staining of Tissue and Cell Sections,” CSH protocols 2008 (2008 1 May): pdb.prot4986, 10.1101/pdb.prot4986. 21356829

[36] Z. Darzynkiewicz , E. Bedner , and P. Smolewski , “Flow Cytometry in Analysis of Cell Cycle and Apoptosis,” Seminars in Hematology 38, no. 2 (2001): 179–193, 10.1016/s0037-1963(01)90051-4. 11309699

[37] T. Inder , B. Darlow , K. Sluis , et al., “The Correlation of Elevated Levels of an Index of Lipid Peroxidation (MDA‐TBA) With Adverse Outcome in the Very Low Birthweight Infant,” Acta Paediatrica 85, no. 9 (1996): 1116–1122, 10.1111/j.1651-2227.1996.tb14228.x. 8888929

[38] Y. Qin , Y. Shi , Q. Yu , et al., “Vitamin B12 Alleviates Myocardial Ischemia/Reperfusion Injury via the SIRT3/AMPK Signaling Pathway,” Biomedicine & Pharmacotherapy 163 (2023): 114761, 10.1016/j.biopha.2023.114761. 37126929

[39] L. Feng , Y. Li , M. Lin , et al., “Trilobatin Attenuates Cerebral Ischaemia/Reperfusion‐Induced Blood‐Brain Barrier Dysfunction by Targeting Matrix Metalloproteinase 9: The Legend of a Food Additive,” British Journal of Pharmacology 181, no. 7 (2024): 1005–1027, 10.1111/bph.16239. 37723895

[40] Y. Zhang , Y. Wang , J. Xu , et al., “Melatonin Attenuates Myocardial Ischemia‐Reperfusion Injury via Improving Mitochondrial Fusion/Mitophagy and Activating the AMPK‐OPA1 Signaling Pathways,” Journal of Pineal Research 66, no. 2 (2019): e12542, 10.1111/jpi.12542. 30516280

[41] Z. L. Yuan , Y. Z. Mo , D. L. Li , L. Xie , and M. H. Chen , “Inhibition of ERK Downregulates Autophagy via Mitigating Mitochondrial Fragmentation to Protect SH‐SY5Y Cells from OGD/R Injury,” Cell Communication and Signaling: CCS 21, no. 1 (August 2023): 204, 10.1186/s12964-023-01211-3. 37580749 PMC10426156

[42] I. Rabinovich‐Nikitin , M. Rasouli , C. J. Reitz , et al., “Mitochondrial Autophagy and Cell Survival Is Regulated by the Circadian Clock Gene in Cardiac Myocytes During Ischemic Stress,” Autophagy 17, no. 11 (2021): 3794–3812, 10.1080/15548627.2021.1938913. 34085589 PMC8632283

[43] R. Xu , W. Wang , and W. Wang , “Ferroptosis and the Bidirectional Regulatory Factor p53,” Cell Death Discovery 9, no. 1 (June 2023): 197, 10.1038/s41420-023-01517-8. 37386007 PMC10310766

[44] Y. J. Tang , Z. Zhang , T. Yan , et al., “Irisin Attenuates Type 1 Diabetic Cardiomyopathy by Anti‐Ferroptosis via SIRT1‐mediated Deacetylation of p53,” Cardiovascular Diabetology 23, no. 1 (April 2024): 116, 10.1186/s12933-024-02183-5. 38566123 PMC10985893

[45] W. L. Lao , Q. L. Song , Z. M. Jiang , W. D. Chen , X. H. Zheng , and Z. H. Chen , “The Effect of Oxycodone on Post‐Operative Pain and Inflammatory Cytokine Release in Elderly Patients Undergoing Laparoscopic Gastrectomy,” Frontiers in Medicine 8 (September 2021): 700025, 10.3389/fmed.2021.700025. 34540861 PMC8440846

[46] Y. Jishi , Y. Hong , and X. Zhongyuan , “Oxycodone Ameliorates the Inflammatory Response Induced by Lipopolysaccharide in Primary Microglia,” Journal of Pain Research 11 (June 2018): 1199–1207, 10.2147/JPR.S160659. 29950892 PMC6018850

[47] M. Kinnunen , P. Piirainen , H. Kokki , P. Lammi , and M. Kokki , “Updated Clinical Pharmacokinetics and Pharmacodynamics of Oxycodone,” Clinical Pharmacokinetics 58, no. 6 (2019): 705–725, 10.1007/s40262-018-00731-3. 30652261

[48] Y. Cen , W. Liao , T. Wang , and D. Zhang , “APPL1 Ameliorates Myocardial Ischemia‐Reperfusion Injury by Regulating the AMPK Signaling Pathway,” Experimental and Therapeutic Medicine 23, no. 2 (2022): 157, 10.3892/etm.2021.11080. 35069838 PMC8753959

[49] J. Cai , X. Chen , X. Liu , et al., “Ampk: The Key to Ischemia‐Reperfusion Injury,” Journal of Cellular Physiology 237, no. 11 (2022): 4079–4096, 10.1002/jcp.30875. 36134582

[50] L. M. Yu , X. Dong , X. D. Xue , et al., “Naringenin Improves Mitochondrial Function and Reduces Cardiac Damage Following Ischemia‐Reperfusion Injury: The Role of the AMPK‐SIRT3 Signaling Pathway,” Food & Function 10, no. 5 (2019): 2752–2765, 10.1039/c9fo00001a. 31041965

[51] K. Au‐Yeung , Y. Shang , C. Wijerathne , S. Madduma Hewage , Y. L. Siow , and K. O , “Acute Kidney Injury Induces Oxidative Stress and Hepatic Lipid Accumulation Through AMPK Signaling Pathway,” Antioxidants (Basel, Switzerland) 12, no. 4 (April 2023): 883, 10.3390/antiox12040883. 37107258 PMC10135179

[52] Y. Shu , D. He , W. Li , et al., “Hepatoprotective Effect of Citrus Aurantium L. Against APAP‐Induced Liver Injury by Regulating Liver Lipid Metabolism and Apoptosis,” International Journal of Biological Sciences 16, no. 5 (January 2020): 752–765, 10.7150/ijbs.40612. 32071546 PMC7019131

[53] L. H. Chien , J. S. Deng , W. P. Jiang , Y. N. Chou , J. G. Lin , and G. J. Huang , “Evaluation of Lung Protection of Sanghuangporus Sanghuang Through TLR4/NF‐κB/MAPK, keap1/Nrf2/HO‐1, CaMKK/AMPK/Sirt1, and TGF‐β/SMAD3 Signaling Pathways Mediating Apoptosis and Autophagy,” Biomedicine & Pharmacotherapy 165 (2023): 115080, 10.1016/j.biopha.2023.115080. 37392658

